# Mid-level managers’ perspectives on implementing isoniazid preventive therapy for people living with HIV in Ugandan health districts: a qualitative study

**DOI:** 10.1186/s12913-024-10803-9

**Published:** 2024-03-08

**Authors:** Canice Christian, Elijah Kakande, Violah Nahurira, Cecilia Akatukwasa, Fredrick Atwine, Robert Bakanoma, Harriet Itiakorit, Asiphas Owaraganise, William DiIeso, Derek Rast, Jane Kabami, Jason Johnson Peretz, Starley B. Shade, Moses R. Kamya, Diane V. Havlir, Gabriel Chamie, Carol S. Camlin

**Affiliations:** 1 University of California San Francisco, San Francisco, CA USA; 2 Infectious Diseases Research Collaboration, Kampala, Uganda; 3 Sustainable East Africa Research Collaboration (SEARCH)-IPT Trial, Mbarara, Uganda; 4 Makerere University, Kampala, Uganda

**Keywords:** TB preventive therapy, Mid-level managers, Health systems

## Abstract

**Background:**

Isoniazid preventive therapy (IPT) works to prevent tuberculosis (TB) among people living with HIV (PLHIV), but uptake remains low in Sub-Saharan Africa. In this analysis, we sought to identify barriers mid-level managers face in scaling IPT in Uganda and the mechanisms by which the SEARCH-IPT trial intervention influenced their abilities to increase IPT uptake.

**Methods:**

The SEARCH-IPT study was a cluster randomized trial conducted from 2017–2021. The SEARCH-IPT intervention created collaborative groups of district health managers, facilitated by local HIV and TB experts, and provided leadership and management training over 3-years to increase IPT uptake in Uganda. In this qualitative study we analyzed transcripts of annual Focus Group Discussions and Key Informant Interviews, from a subset of SEARCH-IPT participants from intervention and control groups, and participant observation field notes. We conducted the analysis using inductive and deductive coding (with a priori codes and those derived from analysis) and a framework approach for data synthesis.

**Results:**

When discussing factors that enabled positive outcomes, intervention managers described feeling ownership over interventions, supported by the leadership and management training they received in the SEARCH-IPT study, and the importance of collaboration between districts facilitated by the intervention. In contrast, when discussing factors that impeded their ability to make changes, intervention and control managers described external funders setting agendas, lack of collaboration in meetings that operated with more of a ‘top-down’ approach, inadequate supplies and staffing, and lack of motivation among frontline providers. Intervention group managers mentioned redistribution of available stock within districts as well as between districts, reflecting efforts of the SEARCH-IPT intervention to promote between-district collaboration, whereas control group managers mentioned redistribution within their districts to maximize the use of available IPT stock.

**Conclusions:**

In Uganda, mid-level managers’ perceptions of barriers to scaling IPT included limited power to set agendas and control over funding, inadequate resources, lack of motivation of frontline providers, and lack of political prioritization. We found that the SEARCH-IPT intervention supported managers to design and implement strategies to improve IPT uptake and collaborate between districts. This may have contributed to the overall intervention effect in increasing the uptake of IPT among PLHIV compared to standard practice.

**Trial registration:**

ClinicalTrials.gov, NCT03315962, Registered 20 October 2017.

## Introduction

Tuberculosis (TB) is a leading cause of death among people living with HIV (PLHIV) globally [1]. Isoniazid Preventive Therapy (IPT), a form of TB preventive treatment (TPT), reduces TB cases and death, even among PLHIV on antiretroviral therapy (ART) with suppressed HIV viral load [2, 3]. However, IPT uptake has remained suboptimal in Sub-Saharan Africa and barriers, including supply issues and knowledge gaps, persist [4, 5].

Multiple studies have focused on and identified provider and patient-level barriers to IPT uptake among PLHIV. Provider-level barriers have included staff shortages, lack of policy-maker support, knowledge gaps, communication challenges with patients, and negative attitude/fears surrounding IPT efficacy, side effects and drug resistance [6–9]. For example, frontline providers in Eritrea described barriers to IPT for PLHIV, including knowledge gaps among providers, particularly around fear of adverse effects of IPT, and inadequate systems for laboratory testing [10]. Patient-level barriers have included knowledge gaps about the benefits of IPT, fear of side effects, pill burden, HIV/TB related stigma, socio-demographic characteristics (i.e. age, educational level, and sex), lack of motivation, and financial burden, including transportation costs and lost time at work due to time spent in clinic [6, 9]. A study in Kampala, Uganda similarly identified insufficient understanding of IPT and fear of side effects as patient-level barriers to IPT [11]. Less is known about district level health system and mid-level management barriers to scaling IPT for PLHIV.

The SEARCH-IPT cluster randomized trial intervened at the level of district health officers: mid-level managers in the health system of Uganda. The SEARCH-IPT intervention included multiple components including (1) bi-annual collaborative meetings with district managers to address challenges to scaling IPT; (2) district-level data feedback at each meeting; (3) leadership and management training for mid-level health managers annually; and (4) an SMS platform to ease communication over a 3-year study period. Mid-level managers in the health system in Uganda oversee health care delivery in their districts. These mid-level managers bridge the gap between Ministry of Health (MoH) guidelines and practical implementation at the district level. Mid-level managers have been shown to play an important role in implementing evidence-based practices globally [12]. The SEARCH-IPT trial (the primary results of which have been previously published) compared an intervention among mid-level managers to standard practice across multiple regions of Uganda. The SEARCH-IPT trial found that the mid-level manager intervention led to an increase in the number of patients started on IPT compared to standard practice, after taking into account national secular trends that influenced IPT implementation [13].

In this analysis, we sought to identify and describe the mechanisms by which the SEARCH-IPT intervention influenced the abilities of mid-level managers to increase IPT uptake in their districts. We also sought to describe shared barriers faced by both intervention and control managers that were not addressed by the SEARCH-IPT intervention.

## Methods

### Study design

The SEARCH-IPT study was a cluster randomized trial, where the unit of randomization was a cluster of districts in Uganda. A cluster randomized trial design was used because the intervention was provided to groups of managers, with one district health manager enrolled per district. The primary results of the trial and further description of the intervention have been published elsewhere [13]. In brief, we enrolled mid-level health managers beginning in November 2017 and follow-up ended in September 2021. The primary outcome of interest was the initiation of IPT among adults with HIV. The intervention was based on the PRECEDE model for behavioral change [14]. Predisposing and reinforcing factors of the intervention were informed by the PRECEDE model for health promotion strategies and included collaborative groups where leadership and management skills were taught through a ‘Mini-MBA’ course over a 3-year period. The trial intervention’s leadership and management training curriculum provided several tools, taught in interactive sessions with coaching by international business professionals and Uganda HIV/TB expert clinicians. These tools were designed to identify challenges managers faced and to develop strategies to overcome these challenges and accomplish their goals. During the meetings, managers were trained on tools for setting short- and long-term objectives and key results (OKRs) which allowed them to set goals and monitor progress and discuss their goals as a group. Study staff shared data on IPT uptake, and other district-level TB metrics, throughout the follow-up period to enable the tracking of progress overtime and comparisons between groups. Mini-collaboratives met every 6-months, with new leadership and management training material taught annually and reinforced at intervening collaborative meetings.

Data for this analysis were derived from transcripts of annual Focus Group Discussions (FGDs), Key Informant Interviews (KIIs), and participant observation field notes. FGDs and KIIs were conducted annually during the SEARCH-IPT intervention, starting at baseline, through three years of follow-up. Mid-level managers in intervention districts participated in FGDs, whereas a sample of managers in control districts participated in KIIs. KIIs were conducted among control districts instead of FGDs to avoid the creation of collaborative groups (similar to the intervention) among control managers. Study staff conducted participant observations at the majority of mini-collaborative meetings during the SEARCH-IPT intervention. There was no patient or public involvement in the design of this study.

### Study population

The study population included all mid-level managers (district health officers and district TB supervisors) who participated in the SEARCH-IPT trial. Details on sample selection in the SEARCH-IPT trial have been published elsewhere [13]. Managers from 25 districts in the Southwest (12 control and 13 intervention districts), 23 districts in the East (11 control and 12 intervention districts), and 34 districts in the East Central (16 control and 18 intervention districts) enrolled. All FGDs included two cadres of mid-level managers from the SEARCH-IPT trial (district health officers [lead manager] and district-level TB supervisors [who report to the district health officer]), except the baseline FGDs in the Eastern region, when a separate FGD was conducted among district health officers and district TB supervisors due to the number of FGD participants. Study staff conducted participant observations at 12 meetings throughout the 3-year follow-up in the southwestern and eastern regions.

### Data collection

FGDs and KIIs were conducted annually. At the FGDs, the number of participants ranged from 7 to 12. A team of trained qualitative researchers collected the data. The qualitative research team was trained on the study material at the beginning of the study and a second refresher training occurred midway through the study. Researchers conducted all FGDs and KIIs in English. All meetings where participant observations occurred were conducted in English; field notes were taken to capture discussions. FGDs and KIIs were audio recorded and transcribed verbatim for analysis.

Topic areas of the FGDs included general feedback on the mini-collaborative meetings, how mini-collaborative meetings compared to other meetings supervisors had attended, the most interesting and challenging components of the meetings and cross-district collaboration, the effects of seeing IPT and TB data compared between districts, challenges with IPT delivery and motivations for making change in their districts. KIIs consisted of semi-structured interviews using open-ended questions. Topics covered in the KIIs included discussion of the burden of TB in their districts, standard TB control practices, challenges related to IPT delivery, changes to TB prevention policy, and motivations for change in their district. Focus groups lasted between 71 – 147 min (average = 111 min) and the KIIs lasted 17 – 76 min (average = 37 min); in the results below, individual participants in FGDs are denoted (‘P1’, ‘P2’, etc.). Study staff developed participant observation field notes from attending and observing mini-collaborative meetings, including notes on general observations of the conduct of the meeting, interactions between participants and study staff, as well as some direct quotes from the participants.

### Data analysis

Dedoose qualitative software was used to analyze data from FGDs, KIIs, and participant observation reports [15]. A framework analysis approach, which falls within the qualitative research tradition of thematic analysis, was used for this study [16]. We used a hybrid approach to code the data, involving both a priori codes that were informed by theory, and focused inductive coding, in which the categories of meaning were derived directly from review of the empirical data. This hybrid approach to coding included the use of established theories for behavior change as well as allowed for the data to speak for itself which allowed for a comprehensive, adaptive approach to analyzing the data. For the development of the initial codes, the first author conducted focused inductive coding on the sample of transcripts (approximately 20% of the data), developed the initial coding framework, then applied the framework to the remainder of the data [17–19]. The a priori codes were informed by a set of theories, including diffusion of innovation theory [20] and social learning theory [21]. Following the inductive coding phase, the first author and last authors developed an analytical framework based on the initial set of transcripts, the sample of data, and categories of grouped codes. The first author then applied the codes to the remaining transcript data using the identified codes and categories. The first and last author developed framework tables for reducing and synthesizing data, as described by Gale (2013), and with subsequent interpretation, including across-case analysis to identify the emergent themes presented here [22].

## Results

Overall, 163 mid-level managers were enrolled from 82 districts, representing 61% of the districts in Uganda. We conducted a total of 6 FGDs and 23 KIIs over the 3-year study period. Four overarching themes emerged from the data when mid-level managers discussed factors that either enabled or impeded them from making positive changes in their districts: agenda setting, collaboration, availability of resources, and motivations (Table 1). When discussing factors that enabled positive outcomes, intervention managers described feeling ownership over interventions, supported by the leadership and management training they had received, and the importance of collaboration. In contrast, when discussing factors that impeded their ability to make changes, intervention and control managers described external funders setting agendas, lack of collaboration in some meetings, inadequate supplies and staffing, and lack of motivation among frontline providers. Quotes and elaboration of these themes are below.

**Table 1:** Key themes and sub-themes identified by mid-level managers in Uganda to implementing changes for IPT uptake in the SEARCH-IPT trial

Themes	Sub-theme
Barriers and enablers identified by intervention managers	
Agenda Setting	• Managers feeling of ownership over local strategies to promote IPT • Power to set agendas impeded by external funders and implementing partners^a^
Collaboration	• Collaborating with other districts enabled sharing of best practices • Positive effect of the pressure of social comparison and recognition • Collaboration within districts between cadres of mid-level managers (district health officers and district tuberculosis supervisors) enabled scaling of IPT • Ministry of Health meetings were top-down and did not facilitate collaboration or district input
Barriers and enablers shared by intervention and control managers	
Availability of resources	• Inadequate INH stocks and inconsistent supplies was a barrier to IPT uptake • Collaboration within districts and with other districts to redistribute available supplies • Frontline provider turnover and knowledge gaps was a barrier for scaling IPT
Motivations	• Managers motivated by improving the health of their constituents • Lack of motivation of frontline providers was a barrier to IPT uptake • Lack of political leadership and prioritization of IPT scale up

^a^Described as an enabler in intervention districts, and as a barrier by control managers

### Agenda Setting

#### Managers feeling of ownership over local strategies to promote IPT

During all FGDs with managers in the intervention group (6 of 6 FGDs), managers mentioned the feeling of ownership over local strategies to promote IPT as motivation for scaling IPT in their districts. Many of these local strategies were developed by managers during collaborative meetings as part of the trial intervention. This manifested in a sense of independence in deciding how change was enacted in one’s district to realize a goal: in this case, the scaling of IPT.

*P8: To me I can say that it is motivational because you can identify the problem yourself. It motivates you to actually look at the problem yourself, and put in place strategies.*

*P7: And you take the charge, you become responsible, you feel it is yours.* – FGD at 2 Year Follow-up with trial intervention district health officers in the Southwest

Managers discussed the expected long-term sustainability of the SEARCH-IPT intervention after trial completion, in part because the intervention promoted the use of available resources and the mid-level managers’ self-motivation to take ownership of local strategies they developed to promote IPT. For example, an intervention manager in the East said, *“This has made us use the resources that we have, which means if [the SEARCH-IPT study] is not to be there, the implementation with IPT would go on.”*

#### Power to set agendas impeded by external funders and their implementing partners

However, managers also felt that the role of external funders and implementing partners counteracted their sense of control and ownership. During 4 of 6 FGDs (67%) and 11 of 23 KIIs (48%), managers discussed the roles that implementing partners play in scaling IPT in their districts, including implementing strategies to improve health, allocating funding, and establishing trainings. This finding contrasted with the intervention managers' discussion of the benefits of independence in deciding how change is enacted in one’s district to realize the goal of scaling IPT. Intervention and control managers described frustration with implementing partners setting the agenda and determining what steps to prioritize to improve IPT implementation, particularly concerning funding allocation and local intervention strategies.

*Some [Implementing Partners] come to support activities, say for TB programs, when as a district, you have other areas that need support because at the district, you know your problems more than these [Implementing Partners] do. But when they come at the district, they tell us that they are going to support this and this program, but we tell them ‘please, me I want support in this and this’. -* FGD at 3 Year Follow-up with trial intervention district health officers and district TB supervisors in Southwest

An intervention manager argued that managers should not always be dependent on the implementing partners: *“If you have issues in your house, you do not expect another man to keep coming to solve them; implementing partners should not be depended on.”* – Participant observation field note at the trial intervention baseline meeting in Southwest Uganda.

Intervention managers also reported challenges associated with losing funding for a particular project or support for activities and how that made it difficult to plan and execute TB prevention and TB treatment.

*You find that when the [Implementing Partner] moves, they move with the program or projects... They tell you that in the next two months, they will not be supporting you in this program because the donors have withdrawn the money and the district does not have the money to take over the program that this [Implementing Partner] has been managing and you find yourself in a mix.* – FGD at 2 Year Follow-up with trial intervention district health officers in Southwest

### Collaboration

#### Collaborating with other districts enabled sharing of best practices

Managers discussed ways in which the intervention helped them to cope with or devise strategies to address challenges. Intervention managers mentioned collaboration with other districts as an enabler to IPT uptake in all FGDs (6 of 6, 100%). This included the discussion of how mini-collaborative meetings in the SEARCH-IPT trial helped with troubleshooting challenges and developing implementation strategies to address IPT uptake.

*You find that your colleagues share best experiences of how they have carried out an activity… It motivates you to say ‘I can also do it.’ If a certain district can perform to this level, then I can also improve.* - FGD at 2 Year Follow-up with trial intervention district health officers in Southwest

#### Positive effect of the pressure of social comparison and recognition

Intervention managers discussed being motivated to improve IPT uptake in their district by comparisons to other districts during the study intervention. During the mini-collaborative meetings, study staff presented dashboards to each district that included quarterly data from the Ministry of Health on TB cases and the number of people with HIV started on IPT. Researchers tracked progress over time and presented an anonymized summary of progress to the group during the meeting, which enabled each district to see how they compared to other districts without sharing which districts fell on the ranking. A manager in an intervention district in the Southwest said, *“If you are there alone, you may think that you are doing very well or you may think that this is not important. But after sitting as a team, you see how [a different district] is doing and you realize that ‘I am sleeping.’”.*

Similarly, control managers discussed wanting feedback to be able to make these comparisons so they could better understand how other districts are faring in the scaling of IPT. A manager in a control district in the Southwest said,* “I would like to have frequent feedback, so that we can be able to know how we stand, because internally I might be thinking that I am doing well in my district when actually my neighbors are doing better, and so eventually I could learn from them.”*

Both intervention and control managers reported being motivated by accountability and recognition as a key driver to scaling IPT. For example, a manager from an intervention district in the Southwest said, *“There is a way you can feel motivated, and you will say, yes I can continue doing this because you are being appreciated; your achievement is being noticed somewhere.”*

#### Collaboration within districts between cadres of mid-level managers (district health officers and district TB supervisors) enabled scaling of IPT

One positive effect of the trial intervention was improved collaboration and alignment of priorities between district health officers (i.e., lead manager in each district) and district TB supervisors (i.e., TB-specific managers that report to lead managers), supported by the leadership and management training provided in the SEARCH-IPT intervention. This improved within-district collaboration and generated buy-in and support from the district health officer for TB-specific manager activities. Participants discussed the importance of this support, resulting in greater ease in addressing inadequate IPT uptake.

*For example, I am a [district TB supervisor]. However, the people that I supervise, I do not have absolute power or authority towards them and say ‘you have not done the work’. But… when a big drum like the [district health officer] talks about something people tend to pick it up... Now that would also give us power as the [district TB supervisors]; you also stand firm because people will hear you because of your boss.* - FGD at 1 Year Follow-up with trial intervention district TB supervisors in East

Control managers also mentioned that the involvement of the district health officer in their district may help make changes in their district. A manager in the East said, *“I feel if the [district health officer] is involved much, the other frontline health workers will be taking my words a bit more seriously.”*

#### Ministry of Health meetings are top-down and did not facilitate collaboration or district input

Additionally, intervention managers discussed the benefits of collaborating with neighboring districts and taking leadership roles at the mini-collaborative meetings. Participants compared the intervention meetings to meetings held by the Ministry of Health and other entities where they perceived meetings to be more directive or “top-down”. An intervention manager in the Southwest said, *“[Ministry of Health meetings], it is do this, do this; it is like a directive. But here [at the collaborative meetings] we look at what had been done and we suggest on how to improve, on how to go and implement.”*

### Availability of resources

#### Inadequate INH stocks and inconsistent supplies was a barrier to IPT uptake

Managers in intervention and control districts frequently mentioned inadequate resources as barriers to IPT uptake, including insufficient drug stock. For example, an intervention manager in the Southwest said, “*The truth is that we created demand. But over time, the supplies we had for adults were nowhere to be seen.”* There was also a discussion about how inadequate stock not only interrupts the prescription of IPT in the near term but also affects the long-term uptake of IPT. When frontline providers remove IPT from regular prescribing practices due to frequent stockouts, they are slow to prescribe it when IPT does become available because it is not what they have been doing routinely.

*If you really know that this [IPT prescribing] is ‘what I am supposed to do’ and you are not interrupted by [INH] stock outs… but when there is a stock out, people [front line providers] again forget that ‘this is what I am supposed to do routinely’. Then the medicine[s] come, [but] people again relax and they are not taking it as part of what they are supposed to do. So, that makes it difficult.* - FGD at 1 Year Follow-up with trial intervention district TB supervisors in East

There were also discussions of challenges with requesting stock from Uganda’s centralized National Medical Stores (NMS) as a barrier to IPT uptake. Clinic staff may not request stock because they are used to a ‘push’ system for certain drugs where the NMS allots drugs to clinics without clinics placing orders. However, the system for IPT is different, as it requires the clinic to order medication (i.e., a “pull” system). Managers suggested training on proper ordering may be beneficial to mitigating this barrier.

*Delivery of [IPT] depends on facilities because it’s a ‘pull’ system with TB drugs and [IPT]. If a facility doesn’t make an order in two months, it will not get the supply… This has come maybe from knowledge gap in ordering. And some facilities are used to the ‘push’ system, like essential drugs, and that’s where the problem is.* - KII at 2 Year Follow-up with a control TB-specific Manger in East

Intervention and control managers also mentioned inadequate funding limited their ability to access clinics due to a lack of transportation and lack of fuel. One intervention manager in the Southwest said, *“Initially we had some programs that were giving out motorcycles, but then the challenge was that when they give you a motorcycle, then fuel becomes an issue.”*

Intervention and control managers also perceived insufficient staffing as a barrier to IPT. One manager in a control district in the East said, “*the challenge in handling TB/HIV is really understaffing.”*

#### Collaboration within districts and with other districts to redistribute available supplies

Intervention and control group managers discussed resourcefulness in redistributing IPT stock to maximize the use of available stock in their district and region. Intervention group managers mentioned redistribution within districts (i.e., between health centers) as well as between districts, reflecting efforts of the SEARCH-IPT trial intervention to promote between-district collaboration, whereas control group managers mentioned redistribution within their districts.

*We also discovered that some facilities with [IPT] were over stocked and this made us to do some re-distribution and take [IPT] to facilities where the consumption was high. –* FGD at 1 Year Follow-up with trial intervention district health officers in East

#### Frontline provider turnover and knowledge gaps was a barrier for scaling IPT

Another perceived resource-related barrier to IPT uptake included gaps in frontline providers’ knowledge of IPT. This included providers lacking confidence in prescribing IPT due to inadequate training or supervision. For example, a control manager in the East said, *“Some health workers didn’t have the knowledge [of IPT] so they would even fear to initiate clients for fear of the side effects.”*

Managers also perceived the turnover of frontline providers, resulting in new replacement frontline providers with inadequate training in prescribing IPT, as a barrier.

*The main challenges we have are experience in the sites where IPT is given, for instance you go to such a site and mentor a number of staff who are working in that clinic, and maybe two, three months down the road, transfers happen and a particular provider has been moved to another facility, and most times you find it is from high to low volume site, meaning that the capacity we will have built in that person goes to another site that is actually not offering this service. So, we tend to have issues of knowledge gaps created by transfers, whereby the trained person has been moved, and the one coming in is not well oriented, meaning that we have to continuously do mentorships in such clinics if we are to keep the standards.* – KII at 2 Year Follow-up with control district TB supervisors in Southwest

### Motivation

#### Managers motivated by improving the health of their constituents

Many intervention and control managers reported being motivated by positive health outcomes in their districts. Participants discussed seeing improvements in patients’ health as a motivation to work hard in their positions. A manager in an intervention district in the Southwest said, *“They come to you in pain, they are crying but by the time they leave you, they are smiling and you see their quality of life improving day by day. That is good enough to make you say that I can even take an extra mile to do this other thing.”*

#### Lack of motivation of frontline providers was a barrier to IPT uptake

In contrast, participants perceived frontline providers’ negative attitudes as a challenge in scaling IPT. Managers said frontline providers are reluctant to be close to contacts of known TB cases and those who may be eligible for IPT due to fear of being infected with TB. A manager from an intervention district in the East said, “*The issues of attitude in case the frontline health workers, [when they] hear about TB, they may not even wish to go near the patient because they think they should first have a mask before they treat, so even if we say this is a TB contact, they even want to disassociate themselves with the contact.*”

#### Lack of political leadership and prioritization of IPT scale up

Lastly, another motivational barrier was a perceived lack of political leadership to scaling IPT. Intervention and control managers discussed the importance of galvanizing politicians, community leaders, and clinic-in-charges to scale IPT. They described the lack of political will as demotivating in their work to improve health outcomes because of feeling unsupported.

*If you have supervisor, in this case I am talking about the political leadership of the local government and they are blinded, they cannot hear. Even if you play the best tune, they cannot dance to it because they are deaf and blind, they do not see what you are seeing and yet they are your supervisors.* - FGD at 1 Year Follow-up with trial intervention district health officers in Southwest

Both intervention and control managers mentioned politics influencing the uptake of IPT, including agenda setting and political will for the scaling of IPT. One control manager in the East said, *“We have a lot of political interference and that is quite discouraging. You want to do things in a certain way and without any reasonable reason, somebody who doesn’t even know what he is talking about wants you to do things just to suit his political interests.”*

## Discussion

This qualitative study provides insights into the perceptions of mid-level managers in the Ugandan health system on the barriers and facilitators to increasing IPT uptake. This study also highlights the mechanisms by which the SEARCH-IPT trial’s intervention may have impacted IPT uptake among PLHIV (the trial’s primary outcome), by comparing perspectives of intervention to control managers who participated in the trial. We found that the SEARCH-IPT intervention, which included leadership and management training, allowed managers to design and implement strategies to improve IPT uptake in their districts, in contrast to the ‘top-down’ approach perceived by managers to be typical of external funders and implementing partners. This leadership and management training may have changed knowledge and behavior among the mid-level managers, similar to other studies that have used the PRECEDE model to improve knowledge and change health outcomes [23]. Other features of the SEARCH-IPT intervention that enabled IPT uptake included collaboration between districts and the positive impact of accountability through comparing performance with other districts at the meetings. In addition, resourcefulness through the strategic redistribution of resources was employed by both intervention and control managers to scale IPT while dealing with inadequate supplies, though intervention managers relied on the cross-district collaboratives created by the SEARCH-IPT intervention, rather than just within-district redistribution as reported by control managers, to address this challenge.

Health managers’ ownership of locally generated implementation strategies was a motivational enabler to scaling IPT, in contrast to a top-down approach of agenda setting. While the evidence of the effects of decentralization on health systems is mixed [24, 25], strengthening district governance and decision-making have shown promise [26–28]. In Uganda, research has demonstrated that mid-level health managers have felt limited in their abilities to make changes [29]. In contrast, the SEARCH-IPT intervention allowed managers to have greater ownership and creativity in developing local strategies to promote IPT, supported by leadership and management training, by using available resources to maximize uptake. Managers discussed that this approach was likely to have lasting effects and potential sustainability, compared to “top down” strategies.

Intervention managers were also motivated by accountability and comparisons between districts at the collaborative meetings. This is consistent with findings from other studies which focus on decentralization and the shifting of accountability and monitoring progress toward mid-level managers [27]. The Community and District-management Empowerment for Scale-up (CODES) study in Uganda focused on aiding districts in identifying health system bottlenecks for child health. The CODES intervention provided data to managers in participating districts to better monitor and address health system challenges and showed positive effects of the intervention on child health outcomes [27]. Accountability and monitoring of data were likely to be key factors in the success of the SEARCH-IPT and CODES studies in Uganda [13, 27].

Managers frequently mentioned the lack of sufficient supplies, including IPT stock, funding for transportation, and inadequate staffing as barriers to scaling IPT in Uganda. These findings are consistent with other initiatives focused on improving district management of health interventions in Uganda [30]. Despite implementing partners providing funding and resources, the managers described frustration in not having control over which implementation strategies were funded. In contrast, the SEARCH-IPT study approach focused on shifting decision-making power to the district managers who were able to develop their own strategies to reach their goals, supported by management training and tools. Managers discussed being aware of deficits in resources available in their districts and felt that having more input into spending would help in addressing these gaps. In addition, the SEARCH-IPT intervention allowed for collaboration among managers from other neighboring districts, aiding managers in identifying solutions to shared challenges, and within shared contexts, among peers.

This study has strengths and limitations. Strengths of this study include the cluster randomized design, breadth of managers enrolled from the majority of districts in Uganda, and duration of follow-up of 3 years. For limitations, this study did not involve stakeholders outside district health officers or district TB supervisors, such as decision-makers from implementing partners, frontline providers or other members of the Ministry of Health outside of mid-level managers. However, we collected data from district health officers and district TB supervisors over three years, providing a breadth of experience working in this space. Additionally, while this study included managers across three regions of Uganda, the results may not be generalizable to other regions in Uganda, or other national health systems. Additionally, there may be limited generalizability of the study results due to the purposeful nature of this qualitative analysis. However, the perspectives of mid-level managers have not been fully captured when evaluating barriers and enablers to IPT in the past [6] and this study is among the first to provide insight into their experiences.

Our findings suggest that involving mid-level health managers in decision-making, supported by leadership and management training, may allow managers to have a greater sense of ownership in achieving goals in their districts. Creating a space for regional collaboration and comparison may galvanize managers to improve outcomes and allow for sharing of successful interventions.

## Conclusion

In Uganda, mid-level managers’ perceptions of barriers to scaling IPT include limited power to set agendas and control funding, inadequate resources, lack of motivation of frontline providers, and lack of political prioritization. We found that the SEARCH-IPT intervention, composed of leadership and management training and inter-district collaborative groups, enabled managers to design and implement strategies to improve IPT uptake, which may have contributed to the overall positive intervention effect in increasing the uptake of IPT among people with HIV.

## Data Availability

Data will be made available upon request to corresponding author.
